# Mining High-Level Imaging Genetic Associations via Clustering AD Candidate Variants with Similar Brain Association Patterns

**DOI:** 10.3390/genes13091520

**Published:** 2022-08-24

**Authors:** Ruiming Wu, Jingxuan Bao, Mansu Kim, Andrew J. Saykin, Jason H. Moore, Li Shen, on behalf of ADNI

**Affiliations:** 1 University of Pennsylvania, Philadelphia, PA 19104, USA; 2 The Catholic University of Korea, Seoul 06591, Korea; 3 Indiana University, Indianapolis, IN 46202, USA; 4 Cedars-Sinai, West Hollywood, CA 90048, USA

**Keywords:** brain imaging genetics, multigraph clustering, Alzheimer’s disease

## Abstract

Brain imaging genetics examines associations between imaging quantitative traits (QTs) and genetic factors such as single nucleotide polymorphisms (SNPs) to provide important insights into the pathogenesis of Alzheimer’s disease (AD). The individual level SNP-QT signals are high dimensional and typically have small effect sizes, making them hard to be detected and replicated. To overcome this limitation, this work proposes a new approach that identifies high-level imaging genetic associations through applying multigraph clustering to the SNP-QT association maps. Given an SNP set and a brain QT set, the association between each SNP and each QT is evaluated using a linear regression model. Based on the resulting SNP-QT association map, five SNP–SNP similarity networks (or graphs) are created using five different scoring functions, respectively. Multigraph clustering is applied to these networks to identify SNP clusters with similar association patterns with all the brain QTs. After that, functional annotation is performed for each identified SNP cluster and its corresponding brain association pattern. We applied this pipeline to an AD imaging genetic study, which yielded promising results. For example, in an association study between 54 AD SNPs and 116 amyloid QTs, we identified two SNP clusters with one responsible for amyloid beta clearances and the other regulating amyloid beta formation. These high-level findings have the potential to provide valuable insights into relevant genetic pathways and brain circuits, which can help form new hypotheses for more detailed imaging and genetics studies in independent cohorts.

## 1. Introduction

Alzheimer’s Disease (AD) is a complex neurodegenerative disorder characterized by continuous cognitive impairment and eventual amyloid plaques, neurofibrillary tangles, and atrophy patterns in the brain [1,2,3]. As the most common type of dementia, AD is responsible for approximately 5.8 million dementia cases in US [4]. AD has a heritability ranging from 60% to 80% estimated from the twin study [5]. The most widely used approach to identify AD genetic basis is to perform a genome-wide association study (GWAS) or GWAS-based meta-analysis on case-control phenotypes. Over 50 AD-related single nucleotide polymorphisms (SNPs) have been identified [6,7].

Many previous AD studies use GWAS and pathway enrichment analysis to explore the genetic basis of the AD diagnosis [3,7,8,9,10,11,12,13,14]. However, these case-control genetic association studies cannot directly reveal the biological pathways from genetic determinants, molecular signatures, and brain traits to cognitive and clinical outcomes. To bridge this gap, brain imaging genetics [15,16,17] is emerging as a new research field, where quantitative traits (QTs) extracted from brain imaging data are used as intermediate phenotypes to study genetics. These imaging QTs have the potential to not only link genetics with disease outcomes but also capture neuropathological heterogeneity of AD [18,19].

Conventional brain imaging genetics studies perform massive pairwise association analyses between each SNP-QT pair. These individual level SNP-QT signals are high dimensional and typically have small effect sizes, making them hard to be detected and replicated. To bridge this gap, some studies attempt to interpret these results on a macroscopic level or derive high-level understandings. For example, Yao et al. used a two-dimensional enrichment analysis to address this challenge, grouping similar brain regions and genes together via a biclustering approach [20]. Yao’s work identified various high-level two-dimensional imaging genetic modules, which were predefined based on the brain transcriptome data from Allen Human Brain Atlas.

In this work, instead of using the knowledge-driven, predefined imaging genetic modules, we propose an alternative data-driven approach to identify high-level imaging genetic patterns. Based on the detailed SNP-QT associations, we develop a graph-cut algorithm to cluster similar SNPs together so that SNPs within the same cluster tend to have similar associations with QTs across the brain. We construct multiple SNP networks based on different similarity measurements. Each similarity network can be viewed as a weighted graph with a specific similarity measure defined as the edge weight. We employ a multigraph clustering method derived from min-max graph cut to discover SNP clusters that take into consideration of all the studied similarity measures. After that, functional annotation is performed for each identified SNP cluster and its corresponding brain association pattern to provide valuable biological insights at a high level.

We applied this pipeline to an AD imaging genetic study, which yielded promising results. For example, in an association study between 54 AD SNPs and 116 amyloid QTs, we identified two SNP clusters with one responsible for amyloid beta clearances and the other regulating amyloid beta formation. These high-level findings have the potential to provide valuable insights into relevant genetic pathways and brain circuits, which can help form new hypotheses for subsequent imaging and genetics studies in independent cohorts.

## 2. Material and Methods

### 2.1. Data Description

Data used in the preparation of this article were obtained from the Alzheimer’s Disease Neuroimaging Initiative (ADNI) database (adni.loni.usc.edu) [21]. The ADNI was launched in 2003 as a public–private partnership, led by Principal Investigator Michael W. Weiner, MD. The primary goal of ADNI has been to test whether serial magnetic resonance imaging (MRI), positron emission tomography (PET), other biological markers, and clinical and neuropsychological assessment can be combined to measure the progression of mild cognitive impairment (MCI) and early AD. For up-to-date information, see www.adni-info.org. In this study, participants (N = 971) include 202 AD, 218 late MCI (LMCI), 296 early MCI (EMCI), and 255 healthy control (HC) subjects. The baseline structural magnetic resonance imaging (MRI) scans, AV45, and FDG positron-emission tomography (PET) scans, genotyping data, demographic information and clinical assessments are downloaded from the ADNI database (adni.loni.usc.edu). Table A1 shows participant characteristics.

### 2.2. Data Preprocessing

The genotyping data were downloaded and analyzed using PLINK v1.90 [22]. We perform quality control using the following criteria: genotyping call rate >95%, minor allele frequency >5%, and Hardy Weinberg Equilibrium $>1.00\times 10^{-6}$. Then, we select 54 risk variants identified by recent AD genome-wide association studies (GWAS) or GWAS meta-analysis [3,6,7]. Table A2 shows the list of risk variants investigated in this study.

Structural MRI scans are processed with voxel-based morphometry (VBM) using the Statistical Parametric Mapping (SPM) software. All scans are aligned to a T1-weighted template image, segmented into gray matter (GM), white matter (WM), and cerebrospinal fluid (CSF) maps, normalized to the standard Montreal Neurological Institute (MNI) space as 2 × 2 × 2 mm${}^{3}$ voxels. The GM maps are extracted and smoothed with an 8mm FWHM kernel. We then extract the average regional GM measurements from 116 regions-of-interests (ROIs) defined by the automated anatomical labeling (AAL) atlas.

Preprocessed F-18 florbetapir (AV45) PET scans are collected and aligned to the Montreal Neurological Institute space as 2×2×2 mm voxels using SPM. Standard uptake value ratio is computed by intensity normalization based on a cerebellar crus reference region. We then extract the average regional AV45 measurements from 116 AAL ROIs.

The (18)F-fluorodeoxyglucose (FDG) PET measurements are also registered into the same MNI space as 2 × 2 × 2 mm${}^{3}$ voxels by SPM. We then extract the average regional FDG measurements from 116 AAL ROIs.

### 2.3. Method Overview

**Figure 1:**
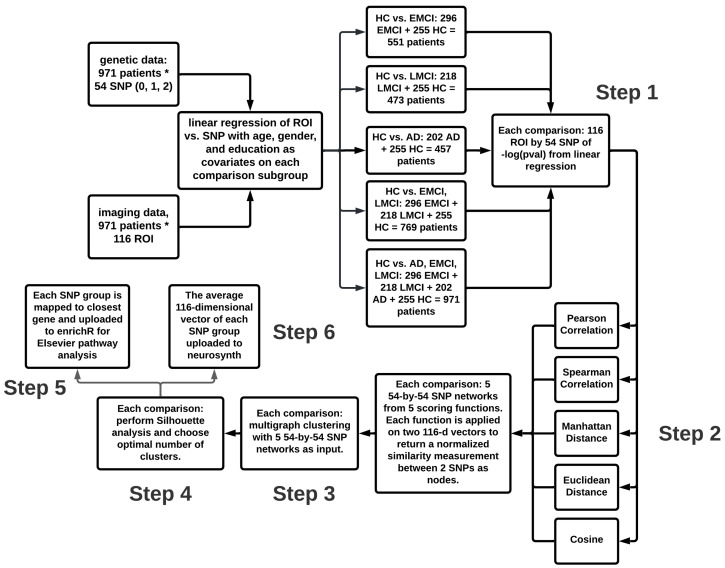
Flowchart of our analysis pipeline. Step 1 generates detailed SNP-QT association maps (54 SNPs by 116 QTs) for five different subject sets examined in our previous study [23], respectively. Step 2 transforms the SNP-QT map to SNP networks by applying different similarity scoring functions to each pair of 116-dimensional SNP vectors. Step 3 uses multigraph min-max cut algorithm to generate an optimal clustering result scoring analysis in Step 4. In Step 5, the SNPs in each cluster are mapped to nearest genes and uploaded to enrichR for Elsevier pathway analysis to identify relevant biological pathways. In Step 6, Neurovault and Neurosynth are used to functionally annotate the average brain association pattern for all the SNPs in each cluster.

Figure 1 shows the flowchart of the analyses performed in this study, including six steps. Step 1 generates detailed SNP-QT association maps for five different subject sets examined in our prior study [23], respectively. Step 2 constructs five SNP similarity networks using different scoring functions. Step 3 performs multigraph clustering on the five SNP networks with a range of cluster numbers. Step 4 examines the clustering quality of each cluster through Silhouette analysis. Based on the Silhouette scoring results, two cluster groups are selected for the subsequent analysis in Steps 5 and 6. We perform functional annotation for (1) each identified SNP cluster in Step 5 using pathway analysis and (2) its corresponding brain association pattern in Step 6 using Neurosynth and Neurovault.

### 2.4. Step 1: Imaging Genetic Association Analysis

The relationship between each ROI-based imaging QT and each SNP can be obtained by performing a linear regression. Let *G* be a set of SNPs and *Y* be a set of imaging QTs (AV45, FDG and VBM). We perform a linear regression model to estimate the additive effect of each SNP g∈G on each QT y∈Y. The analysis is performed for all possible SNP-QT pairs for each of the five comparison groups (i.e., EMCI vs. HC, LMCI vs. HC, AD vs. HC, MCI vs. HC, ALL vs. HC) within each of the three imaging modalities (i.e., AV45, FDG, and VBM). The regression is repeated 54×116 times. The linear regression model is defined as follows:

y=αg+ΓZ+ϵ,

where $Z={\left(z_{1},\cdots ,z_{k}\right)}^{T}$ includes the variables whose effects we want to exclude, such as age, sex, and education; α and $\Gamma =(\gamma_{1},\cdots ,\gamma_{k})$ are the coefficients; ϵ is the error term. Our goal is to estimate α and also test if the SNP *g* has a significant effect (i.e., α≠0) on each QT y∈Y.

Thus, in Step 1 we generate an ROI-based *p*-value map to quantify the significance of SNP effects on imaging data. Specifically, in this work, each element of the significance map records the “negative log *p*-value” $-log_{10}\left(p\right)$ at the corresponding ROI. At the end of this step, we have 5 SNP-QT maps of size 54 (number of studied SNPs) × 116 (number of ROIs) for each of the three modalities.

### 2.5. Step 2: SNP Networks with Different Similarity Measurements

Step 1 explores the lower level relationship between imaging and genetic data. In order to aggregate the individual effects of multiple SNP–ROI pairs to high-level imaging genetic patterns, we transform the SNP-QT maps to an SNP network that models the SNP similarity in terms of their effects on all the QTs across the entire brain. From Step 1, a 54-by-116 SNP-QT map is constructed for each of the five comparison groups within each of the three modalities. For each SNP, there is a 116 dimensional feature representation that maps its effect on the brain. The similarity measurement is applied on all pairs of 116-dimensional normalized SNP vectors to create a 54-by-54 SNP network. Five scoring functions shown in Table 1 are used, resulting in five distinct 54-by-54 SNP networks for each comparison group. The three SNP networks formed by the Pearson correlation, the Spearman correlation, and the cosine similarity are normalized by taking the absolute value of the entry, respectively. The two SNP networks formed by the Manhattan and Euclidean distances are transformed to normalized similarity networks by taking a Gaussian radial basis function centered at distance = 0 with a standard deviation of (maximum–minimum)/3, respectively. After normalization, all the entries in each 54-by-54 SNP network have a value between 0 and 1.

### 2.6. Step 3: Multigraph Min-Max Graph Clustering

Although an SNP network describes the similarity between each pair of SNPs, a high-level understanding can be obtained by grouping similar SNPs together and study their collective effects. From Step 2, five 54-by-54 normalized similarity SNP networks are created for each comparison group within each of the three modalities. The network can be viewed as a graph so that the connected components output from graph cut algorithms are viewed as network clusters. Ding et al. proposed a min-max graph cut algorithm that improves cluster quality and balance by minimizing similarity between pairwise subgraphs and maximizing similarity within each subgraph [24]. The min-max graph cut takes a single similarity network as input, so it clusters one network and examines the effect of one scoring function. Wang et al. generalized the single-graph min-max graph cut into multigraph min-max graph cut, which is used in this study to evaluate the combined effect of five scoring functions [25]. The objective functions of both min-max graph cut models are shown in Table 2. In this study, multigraph min-max graph cut algorithm is implemented through a gradient descent method with convergence conditions. The implication of multigraph min-max clustering is that it combines the effects of multiple scoring functions at the same time. The clustering results of multigraph min-max graph cut algorithm have features that resemble the clustering results of single-graph min-max clustering from the best scoring function. Multigraph min-max clustering with five 54-by-54 SNP networks as inputs is performed on the number of clusters ranged from 2 to 9 to produce clustering results for each comparison group within each modality.

### 2.7. Step 4: Silhouette Scoring Analysis

The goal of this step is to determine the optimal number of clusters. Silhouette refers to a method of interpretation and validation of consistency within clusters of data and provides a graphical representation of cluster quality [26]. The Silhouette value has a range between -1 and 1. A value close to 1 indicates good clustering quality: the objects are close to assigned clusters and far from neighbor clusters. A value close to -1 suggests that the number of clusters selected is not appropriate. The scoring functions are listed in Table 3. The Silhouette scoring analysis is performed on the clustering results of multigraph clustering with number of cluster ranged from 2 to 9. The normalized similarity networks in Step 3 are transformed to distance matrices by converting a similarity measure of *x* into a distance measure of 1−x. For a given number of clusters, there are 5 similarity measurements × 5 comparison groups within each of the three modalities. The 5×5=25 Silhouette scores are averaged for comparison. The clustering result with the highest averaged Silhouette score is selected for further analysis. The Silhouette scoring analysis is also performed on the clustering results of single-graph clustering with number of cluster ranging from 2 to 9. The 5 Silhouette scores from 5 comparison groups are averaged and compared with the averaged Silhouette score of the multigraph clustering to analyze the effectiveness of multigraph clustering.

### 2.8. Step 5: EnrichR Elsevier Pathway Analysis

A high-level result of two SNP groups is produced from previous analysis. The genetic domain of each SNP group can be analyzed through the pathway analysis using Enrichr. Enrichr is an integrative web-based and mobile software application that includes new gene-set libraries, an alternative approach to rank enriched terms, and various interactive visualization approaches to display enrichment results using the JavaScript library, Data Driven Documents (D3) [27,28,29]. The software can also be embedded into any tool that performs gene list analysis. The 54 AD-related SNPs in this study are mapped to their closest gene, upstream or downstream. The SNP cluster from multigraph clustering are mapped to a group of genes and uploaded to EnrichR for pathway analysis. The elsevier pathway analysis results of each SNP cluster are recorded and compared because it contains various AD-related pathways.

### 2.9. Step 6: Neurovault Brain Region Analysis

After analyzing the genetic domain, the brain pattern corresponding to each SNP cluster can be analyzed through mapping the average effect of each SNP group onto the brain. This brain association pattern can be analyzed by Neurovault and Neurosynth [30], which gives us functional and structural information of the affected brain regions. NeuroVault is an open-science neuroinformatics online repository of brain statistical maps atlases and parcellations [30]. Neurosynth is a platform for large-scale, automated synthesis of functional magnetic resonance imaging (fMRI) data. It takes thousands of published articles reporting the results of fMRI studies and outputs brain maps with calculated correlation coefficients given the uploaded MRI data. The SNPs that are grouped together are expected to affect similar brain regions; thus, the averaged SNP effect on 116 QTs from each SNP group is calculated and mapped onto the brain. The resulting brain map is functionally annotated using NeuroVault and Neurosynth.

## 3. Result

### 3.1. Imaging Genetic Association Maps

**Figure 2:**
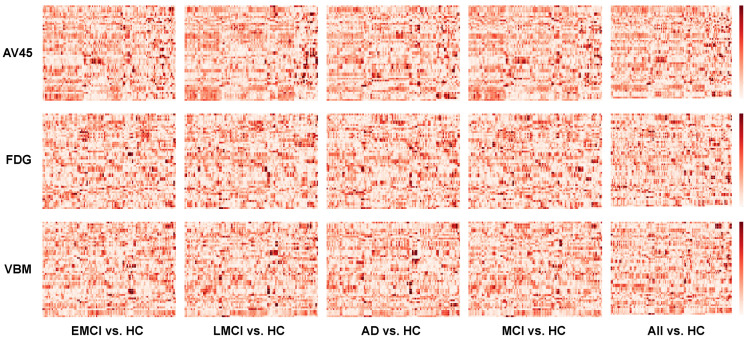
Detailed imaging genetic association maps (54 SNPs by 116 ROIs) with each entry as a normalized $-log_{10}$ (*p*-value) from linear regression of ROI vs. SNP within each comparison group. Normalization was performed so that each row has a squared norm of 1. The vertical axis follows the SNP order listed in Table A2. The horizontal axis follows the ROI order listed in Table A3.

Figure 2 shows all 15 resulting imaging genetic association maps, arranged by three modalities (AV45, FDG, VBM) against five comparisons (EMCI vs. HC, LMC vs. HC, AD vs. HC, MCI vs. HC, All vs. HC). Each map consists of 54 SNPs on the vertical axis and 116 ROIs on the horizontal axis. The order of SNPs on the vertical axis follows the list shown in Table A2. The order of ROIs on the horizontal axis follows the list shown in Table A3.

Each entry of the map corresponds to $-log_{10}$ (*p*-value) from the linear regression before normalization. After an initial SNP-QT map is created, each 116-dimensional vector of a given SNP is normalized such that the Euclidean norm is 1. This step is performed so that each SNP is represented as a directional unit vector to facilitate subsequent analysis.

While such an imaging genetic map describes detailed associations for each SNP-QT pair, it is not straightforward to detect any general trend in these maps. The goal of the subsequent steps is to extract high-level information from these maps and help provide biological interpretation to aid biomarker discovery and therapeutic target identification.

### 3.2. Multigraph vs. Single-Graph Silhouette Analysis

**Figure 3:**
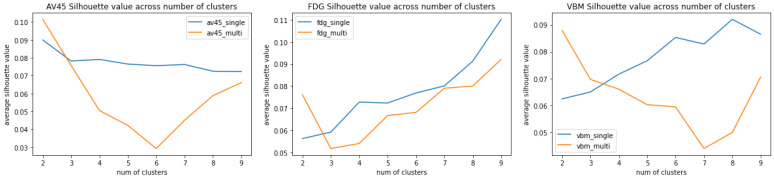
Averaged Silhouette scoring of single-graph and multigraph clustering results across 5 scoring functions × 5 comparison groups at each number of cluster. The results of analyzing AV45, FDG, and VBM data are shown from left to right. In the subsequent analyses, we report the multigraph results of clustering SNPs into two groups, which is the optimal case for both AV45 and VBM.

The multigraph vs. single-graph averaged Silhouette scores are shown in Figure 3. The multigraph averaged Silhouette score is calculated by taking the mean of 25 Silhouette scores (5 scoring functions × 5 comparison groups) from the multigraph clustering result at a given number of clusters for a given modality. The single-graph averaged Silhouette score is calculated by also taking the mean of 5×5=25 Silhouette scores. Instead of using the same clustering result across five scoring functions for the multigraph case, a single-graph clustering is performed on each of the scoring functions. The Silhouette scores are calculated based on the clustering result of a specific scoring function.

A higher Silhouette score indicates a better clustering quality. A lower number of clusters is preferred in this study when the Silhouette scores are similar since our goal is to provide a high-level understanding. As a result, cluster number = 2 is chosen for the subsequent analyses.

### 3.3. Clustering Results

**Figure 4:**
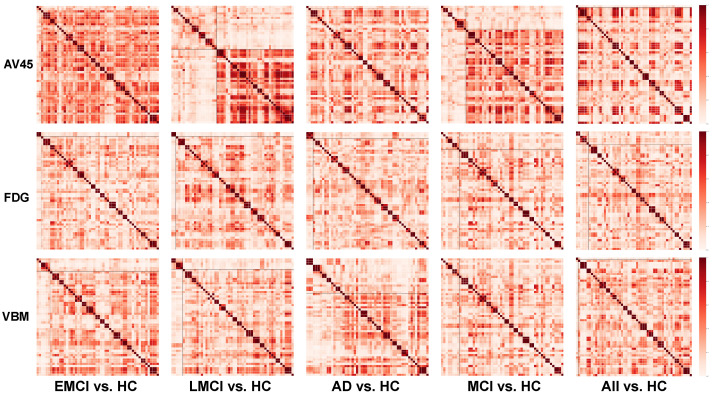
The SNP networks (54 by 54) constructed by the normalized cosine scoring function. Each entry is the cosine similarity of two corresponding SNP representations (measuring their association patterns with 116 ROIs in the brain). The black line indicates the partition of two clusters.

The SNP networks constructed by the normalized cosine scoring function are shown in Figure 4. The two resulting SNP clusters are separated by two black lines. The cluster with a smaller number of SNPs is reordered in the top left corner with the cluster with a larger number of SNPs in the bottom right corner.

The similarity network entries are normalized so that the minimum is 0 and the maximum is 1. Each SNP has a maximum similarity of 1 with itself as observed from the diagonal. Good partition of SNPs is indicated by strong similarity within each cluster and weak similarity between the clusters. A balanced size of the two clusters is preferred so that we can identify multiple high-level patterns instead of one single high-level pattern coupled with a small number of outliers; therefore, the clustering result on the AV45 measures for the LMCI vs. HC comparison group as well as the clustering result on the VBM measures for the AD vs. HC comparison group are selected for subsequent analysis.

### 3.4. Case Study: Example AV45 Result

**Figure 5:**
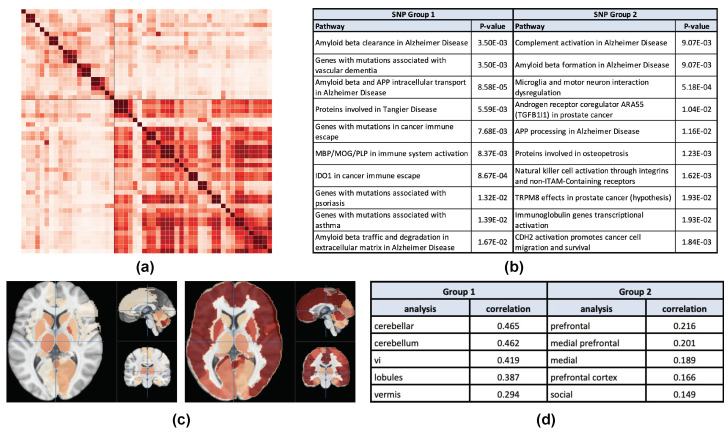
(**a**) Cosine SNP network derived from genetic analysis of the AV45 data in the LMCI vs. HC comparison. (**b**) The Elsevier pathway analysis from EnrichR of SNP group 1 (20 SNPs) and SNP group 2 (34 SNPs). (**c**) The average normalized brain significance maps corresponding to SNP group 1 (left) and SNP group 2 (right), respectively. (**d**) Neurosynth analysis results of the two brain maps shown in (**c**).

Among all the results in modality AV45, the most balanced one is generated by analyzing the LMCI vs. HC comparison group, and this result is shown in Table A4. The functional annotation and pathway analysis of the identified SNP clusters and the corresponding brain maps are shown in Figure 5. The SNPs in each of the two groups are mapped to their closest genes and uploaded as two gene sets to enrichR. The Elsevier pathway analysis is used in this study because multiple AD related pathways are included in this pathway, which is helpful for understanding AD pathogenesis. The average normalized brain significance maps corresponding to two SNP groups are shown in Figure 5c. Neurosynth analysis results of these two brain maps are shown in Figure 5d.

### 3.5. Case Study: Example VBM Result

**Figure 6:**
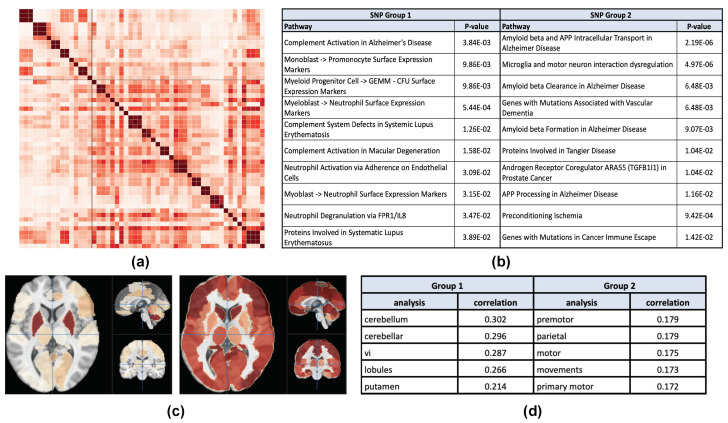
(**a**) Cosine SNP network derived from analyzing VBM data in the AD vs. HC comparison. (**b**) The Elsevier pathway analysis from EnrichR of SNP group 1 (16 SNPs) and SNP group 2 (38 SNPs). (**c**) The average normalized brain significance maps corresponding to SNP group 1 (left) and SNP group 2 (right), respectively. (**d**) Neurosynth analysis results of the two brain maps shown in (**c**).

Among all the results in the modality VBM, the most significant and balanced result is generated by analyzing the AD vs. HC comparison group, and this result is shown in Table A5. The functional annotation and pathway analysis of the identified SNP clusters and the corresponding brain maps are shown in Figure 6. The analysis is similar to the previous case study on the AV45 measures for the LMCI vs. HC comparison group. This clustering result has a lower Silhouette score (0.158) than that in the previous case study (0.293). So a less distinct pattern is observed in the network, along with less differentiated pathways, brain regions, and brain map visualization.

## 4. Discussion

### 4.1. Comparison between Single-Graph and Multigraph Clusterings

In this study, multiple scoring functions have been selected to evaluate the similarity between different AD-related SNPs in terms of their effects on 116 ROIs across the brain. Each scoring function quantifies the similarity between SNPs from a specific perspective. Multigraph clustering is used to output a clustering result that combines the effects of multiple scoring functions. The purpose of building SNP–SNP networks through different scoring methods is to evaluate the SNP similarity in terms of their effects on 116 ROIs traits across the brain from multiple perspectives. Given two vectors (1, 2, 3) and (0.001, 0.002, 0.003), their Pearson correlation, Spearman correlation, and cosine similarity are all 1 (corresponding to the largest similarity), since they focus on comparing the vector directionality instead of the vector magnitude; however, their Manhattan distance and Euclidean distance are very sensitive to the vector magnitude, and thus are both large, leading to very small similarity. Our multigraph approach combines the effects of all these scoring functions, and takes into consideration both vector directionality and magnitude when performing multigraph clustering.

Several single-graph and multigraph clusterings with a varying number of clusters from 2 to 9 are performed. Averaged Silhouette analysis scores are used to quantify clustering quality under a given cluster condition. In Figure 3, the plot of averaged Silhouette analysis for single-graph shows that clustering quality improves in general as the number of clusters increases for FDG and VBM; however, for AV45 a higher number of clusters leads to a lower cluster quality. There is an inconsistency in the optimal number of clusters for different imaging modalities. The goal of this study is to acquire a high-level understanding of imaging genetic associations. Despite the inconsistency of clustering quality, a large number of clusters also makes subsequent analysis complicated. Only a few brain regions and pathways will be present when the number of SNPs in each cluster decreases, which downgrades the high-level understanding back to individual level analysis.

With these difficulties addressed in single graph clustering, the use of multigraph clustering is very promising for various reasons. The first advantage of multigraph clustering is that at a given number of clusters, it is able to selectively use scoring functions that behave well. For example, at cluster number = 2, the Pearson and Spearman methods have low Silhouette scores (<0.062) across all three modalities, while the Manhattan, Euclidean, and cosine methods have high ones (>0.11). In this case, the multigraph clustering yields an average Silhouette score of 0.1016 (Figure 3), resulting in prominent patterns when mapped to Manhattan, Euclidean, and cosine networks (e.g., Figure 5a).

The second advantage of multigraph clustering for this study is that it behaves the best for AV45 and VBM at the number of clusters = 2 (see Figure 3). As discussed above, a small number of clusters is great for high-level analysis. For FDG, the Silhouette score for the cluster number of 2 is also close to the score for the cluster number of 8. So the result for the cluster number of 2 is reported for all three modalities in this study and coupled with subsequent functional annotation and pathway analysis.

The third advantage of multigraph clustering is that the analysis is more efficient and consistent than a collection of single-graph clusterings. Instead of doing five single-graph clusterings with inconsistent results among different scoring functions, multigraph clustering is able to return a single set of clustering result. This feature provides a novel way of analysis for future studies with a large number of candidate evaluation functions and no prior knowledge of their performances.

### 4.2. AV45 Clustering Result

In the AV45 row of Figure 4, comparison group AD vs. HC and ALL vs. HC both have one cluster group of 1 SNP and another cluster group of 53 SNPs. The two clusters can be viewed as one group because the multigraph clustering algorithm explicitly enforces each cluster to be nonempty. While these two results are not significant, rs11278892 with its minor allele G is classified to be the most distant from the other 53 SNPs.

Comparison group EMCI vs. HC has one cluster group of 2 SNPs and another cluster group of 52 SNPs. Again, this can be roughly viewed as a single group. The smaller cluster group contains rs4575098 and rs4663105. There is no prior research of rs4575098, but rs4663105 mapped to BINI gene was identified as having a significant association among APOE ϵ4+ and ϵ4− subjects [31]. Future research can be conducted on the association between rs4575098 and rs4663105 as well as their collective role in early MCI development.

Comparison group LMCI vs. HC has the most balanced cluster group for AV45 with one cluster of 20 SNPs and another cluster of 34 SNPs (with APOE rs429358). The partition will provide us with insights of how two groups of SNPs each plays a different role in the LMCI stage. This finding is promising given that (1) LMCI is the transitional stage between EMCI and AD, (2) there are no significant partitions at EMCI and AD, and (3) there is a significant pattern at LMCI. This suggests a potential stage-specific imaging genetic pattern during AD progression, which warrants further investigation. See Section 4.5 for additional discussion on the functional annotation of this high-level imaging genetic pattern.

### 4.3. FDG Clustering Result

In the FDG row of Figure 4, for the smaller cluster group, EMCI vs. HC group has rs10498633 and rs12881735, LMCI vs. HC group has rs10498633 and rs12881735, and AD vs. HC group has rs6656401, rs2093760, and rs4844610. The MCI vs. HC group has eight SNPs and the ALL vs. HC group has six SNPs. In general, the clustering patterns in the networks do not seem as significant as AV45 and VBM. The Silhouette score of FDG (0.076) is also lower than AV45 (0.102) and VBM (0.0879); yet, there is one observation of the results: rs10498633 present in both EMCI and LMCI smaller cluster groups. Previous studies have shown that rs10498633 in SLC24A4 was significantly associated with anisotropy, total number and length of fibers, including some connecting brain hemispheres [32].

### 4.4. VBM Clustering Result

In the VBM row of Figure 4, comparison group MCI vs. HC has one group of 2 SNPs (rs4236673 and rs9331896) and another group of 52 SNPs. Comparison group ALL vs. HC has one group of 1 SNP (rs9271058) and another group of 53 SNPs. These cases can be viewed as having one group instead of two partitions.

Comparison group EMCI vs. HC has a smaller group of six SNPs: rs10808026, rs7810606, rs10498633, rs12881735, rs12590654, and rs113260531. Comparison group LMCI vs. HC has a smaller group of five SNPs: rs4236673, rs9331896, rs10498633, rs12881735, and rs12590654. The SNPs rs10498633, rs12881735, and rs12590654 lie in the intersection of these two groups, potentially having an impact throughout the MCI stage. As mentioned in the FDG section, rs10498633 is also found to be distant from the other AD-related SNPs for VBM modality, which reinforces its unique role associated with anisotropy in the MCI stage.

Comparison group AD vs. HC has the most balanced cluster result with one group of 16 SNPs and another group of 38 SNPs. This provides us with insights about how the two groups of AD-related SNPs each play a different role in AD patients. Functional annotation of this high-level imaging genetic pattern are discussed in Section 4.6.

### 4.5. AV45 Case Study

In Figure 5a,b, the Elsevier pathway analysis reveals some promising results on our genetic analysis of AV45 measures in the LMCI vs. HC comparison: (1) the pathway of amyloid beta clearance in AD is enriched by genes associated with the SNP Group 1, and (2) the pathway of amyloid beta formation in AD is enriched by genes associated with the SNP Group 2. AD pathogenesis is widely believed to be driven by the production and decomposition of β-amyloid peptide [33]. The disease state of AD is closely related to the solubility and the quantity of β-amyloid. Our pathway analysis suggests that the SNPs in Group 1 have potential to be related to the decomposition of amyloid beta while the SNPs in Group 2 to be related to its production. Since AD is characterized by accumulation of β-amyloid, it warrants further investigation that the SNPs involved here can be studied as suppressors and/or promoters to minimize the amount of β-amyloid present [34].

A relevant observation from our pathway analysis is Group 1’s association with amyloid beta and APP intracellular transport in AD and amyloid beta traffic and degradation in extracellular matrix in AD and Group 2’s association with APP processing. β-amyloid is released by sequential proteolytic processing of the amyloid precursor protein, so the inhibition of APP processing and the excitation of intracellular transport, traffic, and degradation together minimize the accumulation of β-amyloid in the extracellular matrix.

Another indicator of Group 1’s role on β-amyloid is the MBP immunal pathway, which is responsible for amyloid beta degradation [35]. The most correlated pathway of Group 2 is complement activation in AD. Complement proteins are integral components of amyloid plaques and cerebral vascular amyloid in AD patient brains, which can be found at the earliest of amyloid deposition [36]. The complement activation also coincides with the clinical expression of Alzheimer’s dementia. Aside from the two group’s direct associations with β-amyloid, the pathway analysis also shows that AD is correlated with different diseases including Tangier Disease, cancer, psoriasis, and asthma. Previous studies have shown that Tangier Disease is caused by mutations of ABAC1, which is closely related to β-amyloid [37].

In Figure 5c,d, the most correlated brain regions associated with SNP Group 1 include cerebellar, cerebellum, vi, lobules, and vermis (see https://neurosynth.org/analyses/terms/, accessed on 16 June 2022 for definition of these terms). Cerebellar and cerebellum are responsible for motor functions and balance. It is also associated with the visual system. Vermis and some subsequent correlated brain regions are also associated with maintaining posture. So, this group is primarily associated with brain regions that are responsible for balance, motor functions, and visual functions. Group 2 is correlated with prefrontal, medial prefrontal, medial, prefrontal cortex, and social. All these regions control cognitive ability, memory management, and emotional impulse. The affected brain regions and their respective functions of two groups of SNPs show a great difference, demonstrating the promise of our clustering result.

### 4.6. VBM Case Study

Figure 6a,b shows the results of Elsevier pathway analysis on our genetic study of VBM measures in the AD vs. HC comparison. SNP Group 1 is associated with complement activation in AD and various pathways that is associated with the immune system and systematic lupus erythematosus, which is a disease categorized by the immune system attacking its own tissues. SNP Group 2 is associated with amyloid clearance and formation pathways, which has an ambiguous downstream function compared with the AV45 results. Thus previous AV45 result shows a better partition, which can also be verified by visually inspecting the SNP networks and comparing the averaged Silhouette scores (0.1015 vs. 0.0879).

In Figure 6c,d, the brain association pattern corresponding to SNP Group 1 includes cerebellum, cerebellar, vi, lobules, and putamen. Cerebullum and cerebellar govern motor functions and balance (see https://neurosynth.org/analyses/terms/, accessed on 16 June 2022 for definition of these terms). The putamen is involved in learning and motor control, including speech articulation, language functions, and cognitive functions. Similar to the Group 1 result of the AV45 analysis above, this group is associated with balance, motor functions, and visual functions. The brain association pattern corresponding to SNP Group 2, on the other hand, is related to premotor, parietal motor, movements, and primary motor. The primary function of the premotor cortex is to assist in integration of sensory and motor information of the performance of an action. The parietal lobes integrate somatosensory signals and information from different modalities. The difference between the two brain maps in this case is less significant than the AV45 analysis above.

## 5. Conclusions

A data-driven analysis pipeline has been proposed in this work to identify high-level imaging genetic patterns. Based on the detailed SNP-QT associations, we develop a graph-cut algorithm to cluster similar SNPs together so that SNPs within the same cluster tend to have similar associations with QTs across the brain. We construct multiple SNP networks based on different similarity measurements. Each similarity network can be viewed as a weighted graph with a specific similarity measure defined as the edge weight. We employ a multigraph clustering method derived from min-max graph cut to discover SNP clusters that take into consideration of all the studied similarity measures. After that, functional annotation is performed for each identified SNP cluster and its corresponding brain association pattern to provide valuable biological insights at a high level.

Our genetic analysis of the AV45 imaging QTs in the LMCI vs. HC comparison yields a prominent clustering pattern in the cosine SNP network. The pathway analysis shows that the identified SNP Group 1 is associated with amyloid beta clearances while the SNP Group 2 is related to amyloid beta formation. The functional annotation using Neurosynth shows that the brain regions associated with SNP Group 1 are related to motor and balance functions while the brain regions associated with SNP Group 2 are related to memory and cognitive functions. These high-level findings have the potential to provide valuable insights into relevant genetic pathways and brain circuits, which can help form new hypotheses for more detailed imaging and genetics studies in independent cohorts.

## Figures and Tables

**Table 1:** Assume the 54-by-116 genetic-imaging matrix is *X*. Scoring functions are applied to $X_{i}$ and $X_{j}$$\in R^{116}$, 116-dimensional row vectors of *X* that maps the effect of a given SNP to 116 brain regions of interest (ROIs). Assume $X_{ik}$ denotes the *i*-th row and *k*-th column entry of *X*. Note that the Manhattan distance and Euclidean distance need to be transformed to the corresponding similarity measures using a Gaussian radial basis function in the third column.

Measurement	Scoring Function	Normalized Similarity
Pearson correlation	$r\left(i,j\right)=\frac{\sum_{k=1}^{n}\left(X_{ik}-\bar{X_{i}}\right)\left(X_{jk}-\bar{X_{j}}\right)}{\sqrt{\sum_{k=1}^{n}{\left(X_{ik}-\bar{X_{i}}\right)}^{2}{\left(X_{jk}-\bar{X_{j}}\right)}^{2}}}$	|r(i,j)|
Spearman correlation	$\rho \left(i,j\right)=1-\frac{6\sum_{k=1}^{n}{\left(rank\left(X_{ik}\right)-rank\left(X_{jk}\right)\right)}^{2}}{n(n^{2}-1)}$	|ρ(i,j)|
Manhattan distance	$d\left(i,j\right)=\left|\right|X_{i}-X_{j}{\left|\right|}_{1}$	$e^{-0.5{\left(\frac{d\left(i,j\right)-d_{min}}{(d_{max}-d_{min})/3}\right)}^{2}}$
Euclidean distance	$d\left(i,j\right)=\left|\right|X_{i}-X_{j}{\left|\right|}_{2}$	$e^{-0.5{\left(\frac{d\left(i,j\right)-d_{min}}{(d_{max}-d_{min})/3}\right)}^{2}}$
Cosine	$cos\left(i,j\right)=\frac{X_{i}\cdot X_{j}}{\left|\right|X_{i}\left|\right|\cdot \left|\right|X_{i}\left|\right|}$	|cos(i,j)|

**Table 2:** Objective functions of single-graph and multigraph clustering. A is the adjacency matrix, which is equivalent to the similarity network in this study. D is the diagonal matrix of A. Q is the output clustering labels. K is the number of clusters.

Graph Cut Algorithm for Cluster Analysis	Objective Function
Single-graph min-max cut	$min_{Q^{T}Q=I}\Sigma_{k=1}^{K}\frac{q_{k}^{T}Dq_{k}}{q_{k}^{T}Aq_{k}}$
Multigraph min-max cut	$min_{Q^{T}Q=I}\Sigma_{v=1}^{m}\Sigma_{k=1}^{K}\frac{q_{k}^{T}D^{v}q_{k}}{q_{k}^{T}A^{v}q_{k}}$

**Table 3:** Silhouette scoring functions. Let $C_{I}$ be the cluster which node *i* belongs to.

Measure	Calculation
mean distance	$a\left(i\right)=\frac{1}{|C_{I}|-1}\Sigma_{j\in C_{I},i\neq j}d\left(i,j\right)$
mean dissimilarity	$b\left(i\right)=min_{J\neq I}\frac{1}{|C_{J}|}\Sigma_{j\in C_{J}}d\left(i,j\right)$
Silhouette value	$s\left(i\right)=\frac{b(i)-a(i)}{max(a(i),b(i))}$

## Data Availability

The datasets used and analyzed during the study are available in the ADNI LONI repository, https://adni.loni.usc.edu/, accessed on 16 June 2022.

## Abbreviations

The following abbreviations are used in this manuscript:

AD	Alzheimer’s Disease
GWAS	genome-wide association study
SNP	single nucleotide polymorphism
QT	quantitative traits
ROI	region of interest
MRI	magnetic resonance imaging
PET	positron emission tomography
HC	healthy control
EMCI	early mild cognitive impairment
LMCI	late mild cognitive impairment
AV45	F-18 florbetapir
FDG	(18)F-fluorodeoxyglucose
VBM	voxel-based morphometry

## Appendix A

**Table A1:** Participant characteristics.

	HC	EMCI	LMCI	AD	Total
Number of subject	255	296	218	202	971
Age	76.35 ± 6.54	71.78 ± 7.28	74.71 ± 8.39	75.85 ± 7.67	74.48 ± 7.67
Sex (Male/Female)	132/123	167/129	129/89	123/79	551/420
Education (Year)	16.37 ± 2.64	12.12 ± 2.64	16.12 ± 2.94	15.83 ± 2.81	16.13 ± 2.75

The list includes 54 susceptibility loci identified by recent landmark AD genetic studies [3,6,7]. The SNP-QT association maps shown in Figure 2 have a vertical axis that follows the order below.

**Table A2:** Selected AD-related SNPs.

rs-ID	Chromosome	Position	Gene Symbol	rs-ID	Chromosome	Position	Gene Symbol
rs4575098	chr1	161155392	ADAMTS4	rs7920721	chr10	11720308	ECHDC3
rs6656401	chr1	207692049	CR1	rs3740688	chr11	47380340	SPI1
rs2093760	chr1	207786828	CR1	rs10838725	chr11	47557871	CELF1
rs4844610	chr1	207802552	CR1	rs983392	chr11	59923508	MS4A6A
rs4663105	chr2	127891427	BIN1	rs7933202	chr11	59936926	MS4A2
rs6733839	chr2	127892810	BIN1	rs2081545	chr11	59958380	MS4A6A
rs10933431	chr2	233981912	INPP5D	rs867611	chr11	85776544	PICALM
rs35349669	chr2	234068476	INPP5D	rs10792832	chr11	85867875	PICALM
rs6448453	chr4	11026028	CLNK	rs3851179	chr11	85868640	PICALM
rs190982	chr5	88223420	MEF2C-AS1	rs17125924	chr14	53391680	FERMT2
rs9271058	chr6	32575406	HLA-DRB1	rs17125944	chr14	53400629	FERMT2
rs9473117	chr6	47431284	CD2AP	rs10498633	chr14	92926952	SLC24A4
rs9381563	chr6	47432637	CD2AP	rs12881735	chr14	92932828	SLC24A4
rs10948363	chr6	47487762	CD2AP	rs12590654	chr14	92938855	SLC24A4
rs2718058	chr7	37841534	GPR141	rs442495	chr15	59022615	ADAM10
rs4723711	chr7	37844263	GPR141	rs59735493	chr16	31133100	KAT8
rs1859788	chr7	99971834	PILRA	rs113260531	chr17	5138980	SCIMP
rs1476679	chr7	100004446	ZCWPW1	rs28394864	chr17	47450775	ABI3
rs12539172	chr7	100091795	NYAP1	rs111278892	chr19	1039323	ABCA7
rs10808026	chr7	143099133	EPHA1	rs3752246	chr19	1056492	ABCA7
rs7810606	chr7	143108158	EPHA1-AS1	rs4147929	chr19	1063443	ABCA7
rs11771145	chr7	143110762	EPHA1-AS1	rs41289512	chr19	45351516	PVRL2
rs28834970	chr8	27195121	PTK2B	rs3865444	chr19	51727962	CD33
rs73223431	chr8	27219987	PTK2B	rs6024870	chr20	54997568	CASS4
rs4236673	chr8	27464929	CLU	rs6014724	chr20	54998544	CASS4
rs9331896	chr8	27467686	CLU	rs7274581	chr20	55018260	CASS4
rs11257238	chr10	11717397	ECHDC3	rs429358	chr19	45411941	APOE

**Table A3:** Region of interest order. This table includes 116 regions of interest in the brain. The SNP-QT association maps shown in Figure 2 have a horizontal axis that follows the order below.

Index	Name	Index	Name	Index	Name	Index	Name
1	Precentral_L	30	Insula_R	59	Parietal_Sup_L	88	Temporal_Pole_Mid_R
2	Precentral_R	31	Cingulum_Ant_L	60	Parietal_Sup_R	89	Temporal_Inf_L
3	Frontal_Sup_L	32	Cingulum_Ant_R	61	Parietal_Inf_L	90	Temporal_Inf_R
4	Frontal_Sup_R	33	Cingulum_Mid_L	62	Parietal_Inf_R	91	Cerebelum_Crus1_L
5	Frontal_Sup_Orb_L	34	Cingulum_Mid_R	63	SupraMarginal_L	92	Cerebelum_Crus1_R
6	Frontal_Sup_Orb_R	35	Cingulum_Post_L	64	SupraMarginal_R	93	Cerebelum_Crus2_L
7	Frontal_Mid_L	36	Cingulum_Post_R	65	Angular_L	94	Cerebelum_Crus2_R
8	Frontal_Mid_R	37	Hippocampus_L	66	Angular_R	95	Cerebelum_3_L
9	Frontal_Mid_Orb_L	38	Hippocampus_R	67	Precuneus_L	96	Cerebelum_3_R
10	Frontal_Mid_Orb_R	39	ParaHippocampal_L	68	Precuneus_R	97	Cerebelum_4_5_L
11	Frontal_Inf_Oper_L	40	ParaHippocampal_R	69	Paracentral_Lobule_L	98	Cerebelum_4_5_R
12	Frontal_Inf_Oper_R	41	Amygdala_L	70	Paracentral_Lobule_R	99	Cerebelum_6_L
13	Frontal_Inf_Tri_L	42	Amygdala_R	71	Caudate_L	100	Cerebelum_6_R
14	Frontal_Inf_Tri_R	43	Calcarine_L	72	Caudate_R	101	Cerebelum_7b_L
15	Frontal_Inf_Orb_L	44	Calcarine_R	73	Putamen_L	102	Cerebelum_7b_R
16	Frontal_Inf_Orb_R	45	Cuneus_L	74	Putamen_R	103	Cerebelum_8_L
17	Rolandic_Oper_L	46	Cuneus_R	75	Pallidum_L	104	Cerebelum_8_R
18	Rolandic_Oper_R	47	Lingual_L	76	Pallidum_R	105	Cerebelum_9_L
19	Supp_Motor_Area_L	48	Lingual_R	77	Thalamus_L	106	Cerebelum_9_R
20	Supp_Motor_Area_R	49	Occipital_Sup_L	78	Thalamus_R	107	Cerebelum_10_L
21	Olfactory_L	50	Occipital_Sup_R	79	Heschl_L	108	Cerebelum_10_R
22	Olfactory_R	51	Occipital_Mid_L	80	Heschl_R	109	Vermis_1_2
23	Frontal_Sup_Medial_L	52	Occipital_Mid_R	81	Temporal_Sup_L	110	Vermis_3
24	Frontal_Sup_Medial_R	53	Occipital_Inf_L	82	Temporal_Sup_R	111	Vermis_4_5
25	Frontal_Med_Orb_L	54	Occipital_Inf_R	83	Temporal_Pole_Sup_L	112	Vermis_6
26	Frontal_Med_Orb_R	55	Fusiform_L	84	Temporal_Pole_Sup_R	113	Vermis_7
27	Rectus_L	56	Fusiform_R	85	Temporal_Mid_L	114	Vermis_8
28	Rectus_R	57	Postcentral_L	86	Temporal_Mid_R	115	Vermis_9
29	Insula_L	58	Postcentral_R	87	Temporal_Pole_Mid_L	116	Vermis_10

**Table A4:** SNP clustering result on the AV45 measures for the LMCI vs. HC comparison. The SNP and the corresponding closest genes are listed for each resulting cluster or group.

	Group 1			Group 2	
**Index**	**SNP**	**Gene**	**Index**	**SNP**	**Gene**
1	rs4575098_A	ADAMTS4	1	rs6656401_A	CR1
2	rs4663105_C	RP11-138I18.2	2	rs2093760_A	CR1
3	rs6733839_T	RP11-138I18.2	3	rs4844610_A	CR1
4	rs6448453_A	AP001257.1	4	rs10933431_G	SPI1
5	rs9381563_C	RNU6-560P	5	rs35349669_T	CELF1
6	rs2718058_G	FERMT2	6	rs190982_G	MS4A6A
7	rs11257238_C	PVRL2	7	rs9271058_A	MS4A6A
8	rs7920721_G	APOE	8	rs9473117_C	PICALM
9	rs10838725_C	BIN1	9	rs10948363_G	RNU6-560P
10	rs983392_G	BIN1	10	rs4723711_T	FERMT2
11	rs7933202_C	INPP5D	11	rs1859788_A	SLC24A4
12	rs2081545_A	INPP5D	12	rs1476679_C	SLC24A4
13	rs867611_G	CASS4	13	rs12539172_T	SLC24A4
14	rs10792832_A	CASS4	14	rs10808026_A	ADAM10
15	rs3851179_T	CASS4	15	rs7810606_T	KAT8
16	rs10498633_T	HLA-DRB1	16	rs11771145_A	RP11-333E1.1
17	rs12881735_C	AL355353.1	17	rs28834970_C	RP11-81K2.1
18	rs12590654_A	AL355353.1	18	rs73223431_T	CNN2
19	rs113260531_A	EPDR1	19	rs4236673_A	ABCA7
20	rs28394864_A	GPR141	20	rs9331896_C	ABCA7
21			21	rs3740688_G	CD33
22			22	rs17125924_G	RP11-61G19.1
23			23	rs17125944_C	MEF2C-AS1
24			24	rs442495_C	CD2AP
25			25	rs59735493_A	GPR141
26			26	rs111278892_G	EPDR1
27			27	rs3752246_G	PILRA
28			28	rs4147929_A	ZCWPW1
29			29	rs41289512_G	NYAP1
30			30	rs3865444_A	EPHA1
31			31	rs6024870_A	EPHA1-AS1
32			32	rs6014724_G	EPHA1-AS1
33			33	rs7274581_C	PTK2B
34			34	rs429358_C	PTK2B

**Table A5:** SNP clustering result on the VBM measures for the AD vs. HC comparison. The SNP and the corresponding closest genes are listed for each resulting cluster or group.

	Group 1			Group 2	
**Index**	**SNP**	**Gene**	**Index**	**SNP**	**Gene**
1	rs6656401_A	CR1	1	rs4575098_A	ADAMTS4
2	rs2093760_A	CR1	2	rs4663105_C	RP11-138I18.2
3	rs4844610_A	CR1	3	rs6733839_T	RP11-138I18.2
4	rs1859788_A	SLC24A4	4	rs10933431_G	SPI1
5	rs1476679_C	SLC24A4	5	rs35349669_T	CELF1
6	rs12539172_T	SLC24A4	6	rs6448453_A	AP001257.1
7	rs11771145_A	RP11-333E1.1	7	rs190982_G	MS4A6A
8	rs28834970_C	RP11-81K2.1	8	rs9271058_A	MS4A6A
9	rs73223431_T	CNN2	9	rs9473117_C	PICALM
10	rs4236673_A	ABCA7	10	rs9381563_C	RNU6-560P
11	rs9331896_C	ABCA7	11	rs10948363_G	RNU6-560P
12	rs3740688_G	CD33	12	rs2718058_G	FERMT2
13	rs113260531_A	EPDR1	13	rs4723711_T	FERMT2
14	rs3752246_G	PILRA	14	rs10808026_A	ADAM10
15	rs4147929_A	ZCWPW1	15	rs7810606_T	KAT8
16	rs3865444_A	EPHA1	16	rs11257238_C	PVRL2
17			17	rs7920721_G	APOE
18			18	rs10838725_C	BIN1
19			19	rs983392_G	BIN1
20			20	rs7933202_C	INPP5D
21			21	rs2081545_A	INPP5D
22			22	rs867611_G	CASS4
23			23	rs10792832_A	CASS4
24			24	rs3851179_T	CASS4
25			25	rs17125924_G	RP11-61G19.1
26			26	rs17125944_C	MEF2C-AS1
27			27	rs10498633_T	HLA-DRB1
28			28	rs12881735_C	AL355353.1
29			29	rs12590654_A	AL355353.1
30			30	rs442495_C	CD2AP
31			31	rs59735493_A	GPR141
32			32	rs28394864_A	GPR141
33			33	rs111278892_G	EPDR1
34			34	rs41289512_G	NYAP1
35			35	rs6024870_A	EPHA1-AS1
36			36	rs6014724_G	EPHA1-AS1
37			37	rs7274581_C	PTK2B
38			38	rs429358_C	PTK2B
